# A comparison in the ability to detect diabetic retinopathy between fasting plasma glucose and HbA1c levels in a longitudinal study

**DOI:** 10.1002/edm2.196

**Published:** 2020-11-04

**Authors:** Yumi Matsushita, Tetsuji Yokoyama, Norio Takeda, Naotatsu Katai, Natsuyo Yoshida‐Hata, Yosuke Nakamura, Shuichiro Yamamoto, Mitsuhiko Noda, Tetsuya Mizoue, Toru Nakagawa

**Affiliations:** ^1^ Department of Clinical Research National Center for Global Health and Medicine Tokyo Japan; ^2^ Department of Health Promotion National Institute of Public Health Saitama Japan; ^3^ Takeda Eye Clinic Chiba Japan; ^4^ Department of ophthalmology Shimoda Medical Center Shizuoka Japan; ^5^ Hikaru Eye Clinic Saitama Japan; ^6^ Department of ophthalmology Kimitsu Chuo Hospital Chiba Japan; ^7^ Hitachi, Ltd. Hitachi Health Care Center Ibaraki Japan; ^8^ Department of Diabetes, Metabolism and Endocrinology Ichikawa Hospital International University of Health and Welfare Chiba Japan; ^9^ Department of Epidemiology and Prevention National Center for Global Health and Medicine Tokyo Japan

**Keywords:** fasting plasma glucose, HbA1c, longitudinal study, retinopathy

## Abstract

**Aims:**

The relationship between HbA1c and diabetic retinopathy is expected to differ between different races. In this study, we verified which of HbA1c and fasting plasma glucose (FPG) is more effective for detecting the diabetic retinopathy longitudinally in a Japanese population.

**Materials and Methods:**

The study subjects underwent health examinations twice (including eye test and questionnaire of lifestyle and health) in 2008‐2009 (baseline) and in 2012‐2013 (4‐year follow‐up). Both non‐DM and DM patients at baseline were included as the participants. Of these participants, who had not been diagnosed with retinopathy at the baseline survey (n = 2427; 2150 men and 277 women) had eye fundus photographs taken four years later (follow‐up survey). The odds ratios of incidence of retinopathy according to the eight groups of FPG and HbA1c were estimated using multiple logistic regression analysis adjusted for sex and age. Receiver operator characteristic analysis was used to evaluate each value associated with the presence or absence of retinopathy.

**Results:**

The odds ratios (95% confidence intervals) of incidence of retinopathy by HbA1c level categories, in ascending order, were 1.0 (ref.), 5.66 (1.14‐28.26), 1.69 (0.24‐12.04), 3.03 (0.50‐18.28), 1.04 (0.09‐11.59), 4.73 (0.78‐28.69), 4.12 (0.74‐22.85) and 24.47 (5.61‐106.75). For both FPG and HbA1c levels, the odds ratio for the development of retinopathy increased linearly with the increases in the levels FPG and HbA1c, and no clear threshold was observed. The AUC values (SE) for FPG and HbA1c were almost the same, at 0.750 (0.046) and 0.732 (0.048).

**Conclusions:**

It was clarified that the higher the level of FPG and HbA1c was, the higher the incidence of retinopathy after 4 years was. There was no clear threshold. The detection ability of the incidence of retinopathy was almost the same between FPG and HbA1c, suggesting it is possible to detect the risk of retinopathy by HbA1c only.

## Graphical abstract

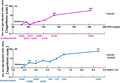

## 1 INTRODUCTION

In recent years, diseases related to lifestyle, such as diabetes, have become an important issue in developed countries and developing countries, with the dramatic transformation of lifestyles due to economic development.^1^

By 2045, the diabetes population will increase by 700 million in the world.^2^ The age‐adjusted prevalence of diabetes all over the world will increase by 9.6% by 2045. With respect to the region, the Western Pacific Region is expected to increase by 12.8% by 2045.

Diabetes causes three major complications: retinopathy, nephropathy and neuropathy. Diabetes also raises the risk of cardiovascular disease.^3^ Of the three major complications of diabetes, retinopathy appears relatively early.

To diagnose diabetes, fasting blood glucose levels are measured twice on different days. The International Advisory Committee, assembled by the American Diabetes Association (ADA), the International Diabetes Federation (IDF) and the European Association for the Study of Diabetes (EASD), recommended one measurement of HbA1c be used as a new diagnostic criterion for diabetes (announced on 5 June 2009 at the symposium of the 69th American Diabetes Association). Currently in Western countries and Japan, diabetes is diagnosed using fasting blood glucose levels and the oral glucose tolerance test. HbA1c is used as an index to reflect the average blood glucose levels of the past 1‐2 months. A study in which HbA1c and the incidence of diabetic retinopathy were observed in a cross‐sectional manner in 960 Pima Indians, 1018 Egyptians and 2821 Americans reported that the risk of onset of diabetes was high when HbA1c was 6.0% or more and less than 6.5%.^3^ Based on this report, the committee commented that HbA1c testing was also useful for the detection of a prediabetic group, and although ‘6.5% should not be an absolute threshold’, ‘people whose HbA1c level is getting close to 6.5% will greatly benefit by taking measures to prevent diabetes’.

The relationship between HbA1c and diabetic retinopathy is expected to differ based on race, but the ability of HbA1c to detect the risk of incidence of retinopathy for Japanese subjects has not yet been determined. In this study, we verified which of the measured values of fasting plasma glucose (FPG) and HbA1c is more effective for detecting the incidence of retinopathy longitudinally in Japanese.

## 2 MATERIALS AND METHODS

### 2.1 Participants

The study design was longitudinal that examined the participants twice at the baseline and four years later. The employees of a company in Ibaraki Prefecture and their spouses underwent health examinations twice (including eye test and questionnaire of lifestyle and health) in 2008‐2009 (baseline) and in 2012‐2013 (4‐year follow‐up), all of which were performed after more than 12 hours of fasting. Both non‐DM and DM patients at baseline were included as the participants. Of these participants, who had not been diagnosed with retinopathy at the baseline survey (n = 2427; 2150 men and 277 women) had eye fundus photographs taken four years later (follow‐up survey).

Informed consent was obtained from each examinee regarding the use of his or her data for research purposes. The present study protocol was approved by the ethics review committee of the National Center for Global Health and Medicine.

### 2.2 Assessment of retinopathy

Retinopathy was assessed with single‐field 45° nonmydriatic fundus photography of each eye using a digital fundus camera (TRC NW300; Topcon Inc). Retinopathy was classified into seven categories, according to the International Clinical Diabetic Retinopathy Disease Severity Scale.^4^ The categories were as follows: (a) no apparent retinopathy, (b) mild nonproliferative diabetic retinopathy, (c) moderate nonproliferative diabetic retinopathy, (d) severe nonproliferative diabetic retinopathy, (e) proliferative diabetic retinopathy, (f) light coagulation and (g) after surgery. We defined subjects with (b)‐(g) as having diabetic retinopathy. If the ophthalmologists know that the subject's FPG and HbA1c levels meet the diagnostic criteria for DM, they may believe more likely to make a diagnosis as retinopathy. To avoid so‐called diagnostic bias,^5^, ^6^ the assessment was done, without prior knowledge of FPG and HbA1c, based on independent grading of the worst eye, by two ophthalmologists specialized in retinal disease. Two ophthalmologists independently examined the fundus images, and another person collected the data. In the instances that the opinions of these two ophthalmologists differed, the ophthalmologists discussed the results again to reach agreement.

### 2.3 Clinical parameters

Height and weight were measured using an automated scale (BF‐220; Tanita) with the patient wearing a light gown. The BMI was defined as the weight (kg) divided by the square of the height (m). Blood was collected from each subject after more than 12 hours of fasting. Blood glucose levels were measured using the glucose electrode technique with an ADAMS glucose GA1170 device (Arkray). HbA1c levels were measured using a HPLC method with an ADAMS HA8160 device (Arkray).

Fasting plasma glucose (FPG) and HbA1c levels at baseline were categorized into eight groups (Group 1, ≤94 mg/dL; Group 2, 95‐99 mg/dL; Group 3, 100‐104 mg/dL; Group 4, 105‐109 mg/dL; Group 5, 110‐117 mg/dL; Group 6, 118‐125 mg/dL; Group 7, 126‐144 mg/dL; and Group 8, ≥145 mg/dL for FPG; and Group 1, ≤5.5%; Group 2, 5.6%‐5.8%; Group 3, 5.9%‐6.1%; Group 4, 6.2%‐6.4%; Group 5, 6.5%‐6.7%; Group 6, 6.8%‐7.0%; Group 7, 7.1%‐7.3%; and Group 8, ≥7.4% for HbA1c).

### 2.4 Data analysis

The incidence of retinopathy, regardless of the status of diabetes at the follow‐up survey, in the eight groups of FPG and HbA1c, is shown. Odds ratios (95% confidence intervals (CIs)) of the incidence of retinopathy according to the eight groups of FPG and HbA1c were estimated using multiple logistic regression analysis adjusted for sex and age.

Receiver operator characteristic (ROC) analysis was used to evaluate each FPG and HbA1c associated with the incidence of retinopathy. ROC analysis is a formal method that plots sensitivity against 1‐specificity to assess the trade‐off between sensitivity and specificity at various test cut‐off points or thresholds, providing a measure of diagnostic accuracy called area under the curve (AUC). We drew the ROC curves for each FPG and HbA1c, and calculated the corresponding AUC. All analyses were performed using SPSS for Windows version 15.0 (SPSS) and Stata 10 (StataCorp LP).

## 3 RESULTS

The characteristics of the subjects at baseline are shown in Table 1. The mean FPG and HbA1c were 105.5 mg/dL and 5.5％, including non‐DM and DM subjects.

**TABLE 1:** Characteristics of the subject

	Mean	SD
n (Men/Women）	2427 (2150/277)	
Age (y)	55.6	7.1
Systolic blood pressure (mm Hg)	121.9	12.3
Diastolic blood pressure (mm Hg)	78.1	8.0
BMI (kg/m^2^)	23.9	2.9
Fasting plasma glucose (mg/dL)	105.5	19.1
HbA1c, NGSP (%)	5.5	0.7

**FIGURE 1:**
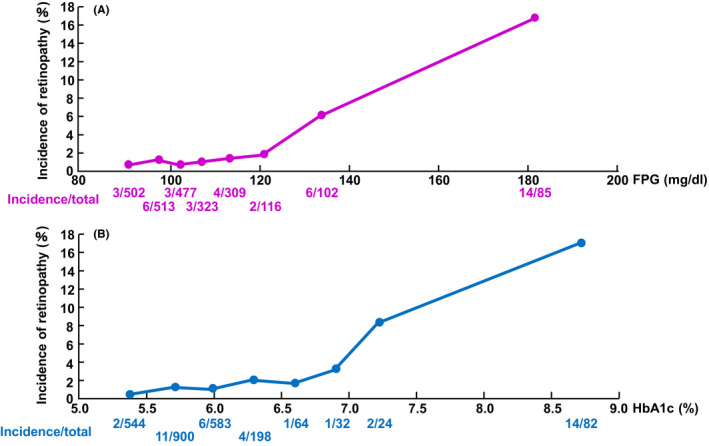
(A) The incidence according to the levels of fasting plasma glucose. (B) The incidence according to the levels of HbA1c. The *x*‐axis is the average of each group

The incidence of retinopathy is shown by blood glucose level in Figure 1A and by HbA1c level in Figure 1B. The number of patients who developed retinopathy during these 4 years was 41 among 2427 (1.7%). It was shown that the higher the levels of blood glucose and HbA1c were, the higher the incidence of retinopathy was.

**FIGURE 2:**
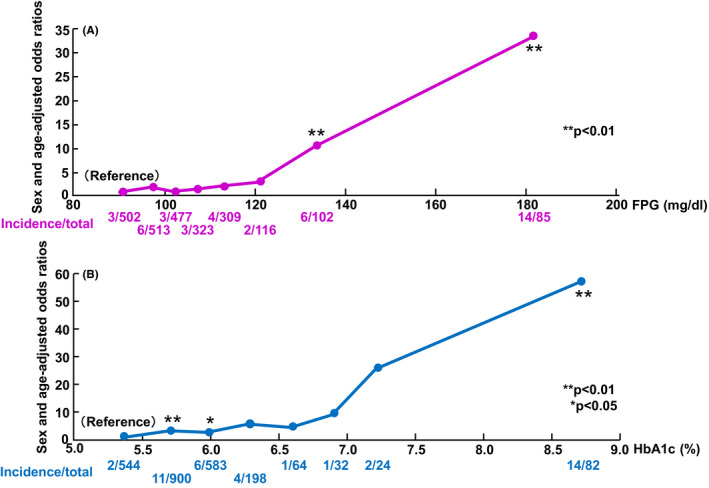
(A) The incidence of diabetic retinopathy according to the levels of fasting plasma glucose. (B) The incidence of diabetic retinopathy according to the levels of HbA1c. The *x*‐axis is the average of each group

**FIGURE 3:**
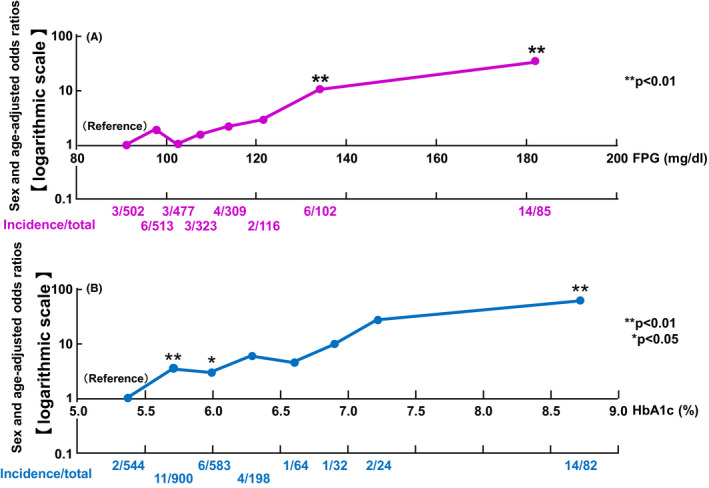
(A) The log‐translated sex‐ and age‐adjusted odds ratio of incidence of diabetic retinopathy according to the levels of fasting plasma glucose. (B) The log‐translated sex‐ and age‐adjusted odds ratio of incidence of diabetic retinopathy according to the levels of HbA1c. The *x*‐axis is the average of each group

The incidence and odds ratios for retinopathy according to the FPG and HbA1c levels are shown in Figure 2A,B. For diabetic retinopathy, the odds ratios (95% CIs) of the FPG level categories were 1.0 (ref.), 1.98 (0.49‐7.99), 1.06 (0.21‐5.32), 1.58 (0.31‐7.90), 2.21 (0.49‐10.05), 2.96 (0.48‐18.07), 10.55 (2.55‐43.64) and 33.23 (9.21‐119.87). The odds ratios of incidence of retinopathy by HbA1c level categories were 1.0 (ref.), 5.66 (1.14‐28.26), 1.69 (0.24‐12.04), 3.03 (0.50‐18.28), 1.04 (0.09‐11.59), 4.73 (0.78‐28.69), 4.12 (0.74‐22.85) and 24.47 (5.61‐106.75). The incidence and sex‐ and age‐adjusted odds ratios for retinopathy increased with increasing FPG and HbA1c levels. It seems that the odds ratios markedly increase in the top two categories of the levels of FPG and HbA1c. To examine if there are the thresholds levels of FPG and HbA1c for the development of retinopathy, odds ratios adjusted for sex and age were plotted in a logarithmic scale. As a result, for both FPG and HbA1c levels, the odds ratio for the development of retinopathy increased linearly with the increases in the levels FPG and HbA1c, and no clear threshold was observed (Figure 3A,B).

**FIGURE 4:**
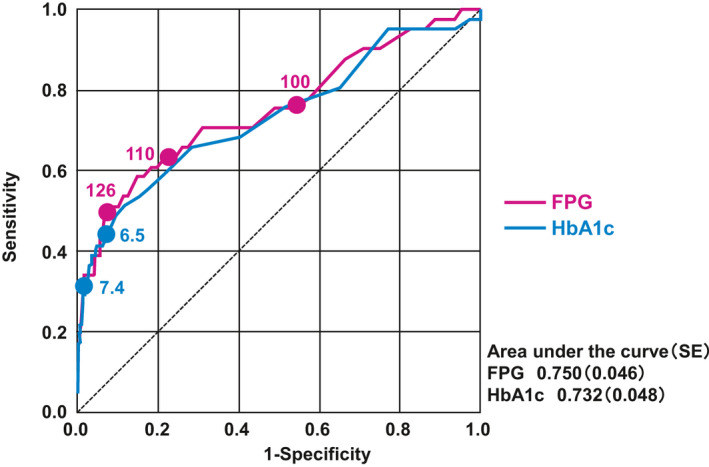
The receiver operator characteristic curves to detect retinopathy

We plotted the ROC curves to compare the ability of FPG and HbA1c in relation to the detection of incidence of retinopathy (Figure 4). The AUC values (SE) for FPG and HbA1c were almost the same, at 0.750 (0.046) and 0.732 (0.048), respectively.

## 4 DISCUSSION

We clearly showed that the incidence and sex‐ and age‐adjusted odds ratios for retinopathy increased with increasing FPG and HbA1c levels without clear threshold. Both FPG and HbA1c had almost the same predictive ability to detect the incidence of retinopathy.

Several studies have compared the sensitivity and specificity of FPG and HbA1c in diabetic retinopathy detection. Many cross‐sectional studies conducted in China, Iran, Korea, the United States, Pima peoples, etc, have reported that HbA1c was able to detect diabetic retinopathy more accurately.^7^, ^8^, ^9^, ^10^ A meta‐analysis also indicated the superiority of HbA1c to FPG for detecting diabetic retinopathy; the odds ratio was 16.32 for HbA1c and 4.87 for FPG, and the area under the ROC curve was 0.837 for HbA1c and 0.735 for FPG.^11^ In contrast, there are only two studies that reported FPG as a better indicator than HbA1c.^12^ In our longitudinal study, the detection ability for retinopathy between FPG and HbA1c was the same.

There have been three longitudinal studies that have used the incidence of retinopathy as the end‐point so far. The first is a 5‐year follow‐up study in 960 Pima Indians that examined diagnostic value of two‐hour postprandial plasma glucose, fasting plasma glucose (FPG) and HbA1c levels using the incidence of retinopathy as the end‐point. Subjects receiving insulin or oral hypoglycaemic treatment at the baseline examination were excluded from the analysis. None of the three indicators had a significant advantage for detecting incident cases of retinopathy.^13^ The second is a 5‐year follow‐up study in 2605 nondiabetic Japanese subjects. It is concluded that Hba1c value of 6.5% (48 mmol/mol) and FPG value of 7.0 mmol/L might be proper as diabetes diagnostic thresholds that indicate a high risk of future retinopathy.^14^ The third is a 3‐year follow‐up study in 21 137 Japanese subjects that assessed the validity of the HbA1c level of 6.5% as the detection threshold of retinopathy.^15^ The longitudinal analysis demonstrated that a HbA1c level of 6.5% was valid as the threshold, but no threshold HbA1c level was found in the cross‐sectional analysis. This study focused only on HbA1c, and validity of HbA1c and FPG levels was not compared. Our study found no threshold for detecting the incidence of retinopathy, although it is difficult to directly compare the result of our study with that of the previous studies because these studies used different methods for diagnosing retinopathy.

This study has several strengths and limitations. As one of its strengths, the sample size of our study was sufficiently large (more than 2400 subjects), and both sexes were included in a longitudinal setting. To our knowledge, no other studies have compared the strength of the detection ability of incidence of diabetic retinopathy between HbA1c and glucose for Japanese. Our study is the first to clarify the risk of each disease by calculating comparable odds ratios longitudinally. Secondly, we used two independent ophthalmologists who specialized in retinal disease who had no prior knowledge of HbA1c or glucose levels to avoid diagnostic bias. Thirdly, our study is thought to have a small measurement bias because eye fundus photographs were measured using the same machine both the baseline examination and the 4‐year follow‐up examination.

The limitations of this study include the use of single‐field retinal photography for measuring diabetic retinopathy. However, a comparison of sevenfold standard stereoscopic fundus photographs, single‐field standard stereoscopic fundus photographs and single‐field nonmydriatic retinal photography showed perfect agreement for identifying people with and without diabetic retinopathy and moderate agreement for retinopathy grading.^16^

It was clarified that the higher the level of FPG and HbA1c was, the higher the incidence of retinopathy after 4 years was, and there was no clear threshold. In this study, we compared the ability to detect retinopathy between FPG and HbA1c using ROC curves in a longitudinal study. The detection ability was almost the same between FPG and HbA1c, suggesting it is possible to detect the risk of retinopathy by HbA1c only. From the viewpoint of the prevention of retinopathy, it became clear that it would be better to keep HbA1c at a low level within a range of not causing hypoglycaemia.

## CONFLICT OF INTEREST

No potential conflicts of interest relevant to this article were reported.

## AUTHOR CONTRIBUTIONS

The principal investigator is Yumi Matsushita, National Center for Global Health and Medicine. Yumi Matsushita takes full responsibility for the work as a whole, including the study design, access to data, and the decision to submit and publish the manuscript. YM, SY and TN researched the data. YM, TY, NT, NK, NY, YN, SY, MN, TM and TN contributed to discussions. YM wrote the manuscript. YM, TY, NT, NK, NY, YN, SY, MN, T.M and TN reviewed and edited the manuscript.
